# The prognostic significance of long noncoding RNAs in non-small cell lung cancer: a meta-analysis

**DOI:** 10.18632/oncotarget.13956

**Published:** 2016-12-15

**Authors:** Wei Jing, Nandi Li, Yingchao Wang, Xuefang Liu, Shengjun Liao, Hongyan Chai, Jiancheng Tu

**Affiliations:** ^1^ Department of Laboratory Medicine, Clinical Laboratory Medicine and Center for Gene Diagnosis, Zhongnan Hospital of Wuhan University, Wuhan, China

**Keywords:** non-small cell lung cancer, long noncoding RNAs, prognosis, lymph node metastasis, meta-analysis

## Abstract

Non-small cell lung cancer (NSCLC) is the most common type of lung cancer. The overall 5-year survival rate of patients is extremely low and to find a new marker is urgently needed. Numerous studies indicate that long noncoding RNAs (lncRNAs) abnormally express in cancers. However, the results have been disputed, especially in the aspects of tumor prognosis. Therefore, we performed this meta-analysis to systematically summarize the relationship between lncRNAs expression and NSCLC. A total of 34 eligible studies including 30 on overall survival, 10 on progression-free survival and 23 on clinicopathological features were identified from the databases. Our results indicated that the levels of lncRNAs were associated with the overall survival (OS; hazard ratios [HR], 1.43; 95% confidence interval [95% CI], 1.17-1.76; *P* < 0.001). However, there was no relationship between lncRNAs and progression-free survival (PFS; hazard ratios [HR], 1.55; 95% confidence interval [95% CI], 0.91-2.63; *P* = 0.11). Moreover, lncRNAs were related to lymph node metastasis (odds ratios [OR], 1.70; 95% confidence interval [95% CI], 1.03-2.80; *P* = 0.04), while no association was observed with other characteristics. In conclusion, our present meta-analysis indicated that lncRNAs transcription levels may serve as a promising marker for prognosis of patients with NSCLC.

## INTRODUCTION

Lung cancer is one of the most common malignant cancers worldwide, making up 18.2% of all malignant tumor mortality in the world [1, 2]. Non-small cell lung cancer (NSCLC) is the most common type, accounting for about 85% of lung cancer [3]. To date, despite the effective treatments for patients have obtained, including surgical resection, systemic chemotherapy and targeted drugs, the prognosis of NSCLC remains still poor and the 5-year survival rate is only 11-15%, because the patients were diagnosed at advanced stages [4–6]. Metastasis and recurrence are the major causes of morbidity and death in NSCLC[7]. Lung cancer is a multi-factor, multi-step and complex process, which was related to many tumor-related genes[8]. In NSCLC researches, several genetic biomarkers have been used, including non-coding RNAs (ncRNAs). Therefore, to identify sensitive and specific biomarkers for prognosis of patients with NSCLC is urgently needed.

Recently, advanced techniques discovered that more than 90% of the mammalian genome can be transcribed into short or long noncoding RNAs[9]. Long non-coding RNAs (lncRNAs) are defined as noncoding RNAs longer than 200 nucleotides with no protein-coding capacity [10]. Increasing evidences have pointed that lncRNAs play tremendous roles in epigenetics and cytobiological processes, including pre-transcription, post-transcription and cell proliferation, differentiation, apoptosis, and migration [9, 11, 12]. Aberrantly expressed lncRNAs have been found in various cancers. For example, the TGF-β-activated lncRNA (lncRNA-ATB), is significantly increased in hepatocellular carcinoma and associated with poor prognosis [13]. In 2016, Han *et al* [10] found that circulating long noncoding RNA GAS5 could be served as a biomarker in breast cancer to assess the surgical effects. In gastric cancer, knockdown of lncRNA X-inactive specific transcript (XIST) could reduce the mRNA and protein levels of EZH2 and suppress the colony formation and invasion ability stimulated by miR-101[14]. Meanwhile, recent studies have shown that lncRNAs could serve as potential diagnostic and prognostic biomarkers for NSCLC.

To date, there are some lncRNAs have been confirmed to aberrantly expressed in NSCLC, such as MALAT1[15], H19[16], and SPRY4-IT1[17]. In 2016, Lu *et al* [18] elaborated that there was a positive correlation between the expression levels of CDKN2B antisense RNA 1 (ANRIL) and c-Myc, and decreased ANRIL significantly inhibited NSCLC cell proliferation. BRAF activated non-coding RNA (BANCR) actively functioned as a regulator of epithelial-mesenchymal transition (EMT) through inducing E-cadherin expression, and decreasing N-cadherin, Vimentin. The results suggested that BANCR could be a biomarker for poor prognosis of NSCLC [19]. CAR intergenic 10 (CAR10) a novel lncRNA, could bind and stabilize transcription factor Y-box-binding protein 1 (YB-1), leading to up-regulation of the epidermal growth factor receptor (EGFR) and proliferation of lung cancer cells, and CAR10-YB-1 represented a potential therapeutic target in NSCLC[11]. Numbers of researches were done to explore the prognostic value of lncRNAs in NSCLC. However, because the sample size and research programs, single study may be inaccurate and insufficient. Thus, we conducted a meta-analysis to evaluate the value of lncRNAs in the prognosis and clinical outcomes in NSCLC through larger sample size of patients.

## RESULTS

### Study characteristics

**Figure 1:**
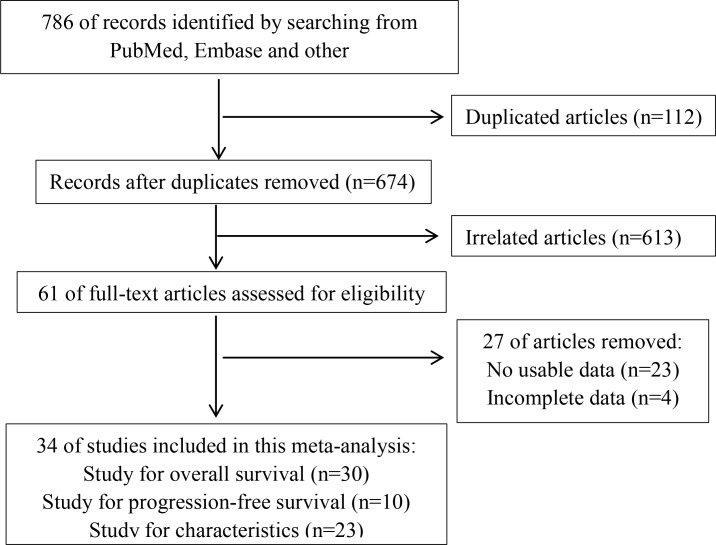
The flow diagram indicated the process of study selection

We searched 786 articles in the databases, as shown in the flow diagram (Figure 1). 725 records were removed after screening the titles and abstracts. Then because of no usable data or incomplete data, 27 papers were excluded. As a result, a total of 34 articles were in the current meta-analysis [3, 5, 16, 17, 19–48], including 30 on overall survival (OS) [3, 5, 16, 17, 19–43, 48], 10 on progression-free survival (PFS) [17, 19, 20, 25, 36, 37, 44–47] and 23 on clinicopathological features[5, 16, 17, 19, 20, 22–28, 30, 32–40, 46].

Table 1 summarized the main characteristics of the included 34 studies ranging from 2003 to 2016, with a maximum sample size of 192 and a minimum sample size of 38 patients. Among these studies, 32 were from China, 1 from Japan and 1 from Germany. Because the cut-off definitions were various, the cut-off values were different in these studies. Meanwhile, in the 34 studies, 23 articles performed the relationship between the expression of lncRNAs and gender[5, 16, 17, 19, 20, 22–28, 30, 32–40, 46], 16 studies referred to the smoking history[5, 16, 17, 19, 20, 22, 23, 25, 30, 32–34, 36, 38–40], 17 studies demonstrated that lncRNAs were correlated with histological classification[16, 17, 19, 20, 22–27, 30, 32–34, 36, 37, 46], 12 studies were on tumor size[16, 17, 19, 20, 22, 25, 30, 32, 33, 36–38], and 11 were about tumor-node-metastasis (TNM) [16, 17, 19, 20, 22, 25, 32, 34, 36–38], while 22 studies reported that lncRNAs were significantly correlated with lymph node metastasis (LNM)[5, 16, 17, 19, 20, 22, 24–28, 30, 32–40, 46] (Table 2).

**Table 1:** Characteristics of studies included in this meta-analysis

Author	Year	Country	LncRNAs	Tumor type	Method	Case number (High/Low)	Outcome	Cut-off	Follow-up months
Hou	2014	China	Sox2ot	NSCLC	qRT-PCR	47	OS	Top quartile	60
Deng	2015	China	AFAP1-AS1	NSCLC	qRT-PCR	66/55	OS	NA	60
Li	2016	China	AGAP2-AS1	NSCLC	qRT-PCR	40/40	OS, PFS	Mean	40
Luo	2014	China	CARLO-5	NSCLC	qRT-PCR	29/33	OS	Median	80
He	2016	China	CASC2	NSCLC	qRT-PCR	38/38	OS	Median	60
Sun	2014	China	BANCR	NSCLC	qRT-PCR	53/60	OS, PFS	Fold-change	40
Han	2013	China	GAS6-AS1	NSCLC	qRT-PCR	25/25	OS	Mean	60
Zhang	2016	China	H19	NSCLC	qRT-PCR	25/25	OS	Median	60
Lin	2015	China	ANRIL	NSCLC	qRT-PCR	48/39	OS	Mean	60
Zang	2016	China	Linc01133	NSCLC	qRT-PCR	34/34	OS, PFS	Mean	40
Xie	2014	China	HMlincRNA717	NSCLC	qRT-PCR	49/69	OS	Mean	80
Zhao	2016	China	SBF2-AS1	NSCLC	qRT-PCR	80/94	OS	Median	70
Wang	2015	China	UCA1	NSCLC	qRT-PCR	36/24	OS	NA	80
Lu	2013	China	MEG3	NSCLC	qRT-PCR	21/21	OS	Median	60
Fang	2016	China	XIST	NSCLC	qRT-PCR	38/15	OS	2.58-fold	NA
Han	2015	China	PANDAR	NSCLC	qRT-PCR	70/70	OS	Mean	60
Nie	2014	China	MVIH	NSCLC	qRT-PCR	21/21	OS	Median	40
Sun	2016	China	NEAT1	NSCLC	qRT-PCR	67/29	OS	2 folds	40
Zhang	2014	China	TUG1	NSCLC	qRT-PCR	96/96	OS	Median	60
Yang	2014	China	PVT1	NSCLC	qRT-PCR	65/17	OS	Median	60
Wan	2016	China	PVT1	NSCLC	qRT-PCR	56/49	OS, PFS	Median	40
Sun	2014	China	SPRY4-IT1	NSCLC	qRT-PCR	60/61	OS, PFS	Median	40
Wang	2016	China	TUSC7	NSCLC	qRT-PCR	56/56	OS, PFS	Median	80
Nie	2016	China	UCA1	NSCLC	qRT-PCR	39/73	OS	Youden index	80
Zhang	2014	China	ZXF1	NSCLC	qRT-PCR	43/19	OS	Ratio=2	36
Yang	2015	China	ZXF2	NSCLC	qRT-PCR	20/20	OS	Median	39
Ji	2003	China	MALAT1	NSCLC	qRT-PCR	70/70	OS	Median	100
Zhang	2015	China	Linc01133	LSCC	qRT-PCR	39	OS	Median	60
Schmidt	2011	Germany	MALAT1	LSCC	qRT-PCR	NA	OS	NA	166
Wang	2015	China	Linc01207	LAD	qRT-PCR	49/11	OS	Fold of 5.78	75
Cheng	2015	China	UCA1	NSCLC	qRT-PCR	20/32	PFS	2-ΔCt =0.068	25
Pan	2016	China	BC087858	NSCLC	qRT-PCR	12/26	PFS	2-ΔCt = 0.142	40
Nakagawa	2013	Japan	HOTAIR	NSCLC	qRT-PCR	17/60	PFS	2-fold	50
Liu	2013	China	HOTAIR	LAD	qRT-PCR	22/19	PFS	HSCORE=74.2	15

LncRNA: Long non-coding RNA; NSCLC: Non-small cell lung cancer; LSCC: Lung squamous cell carcinoma; LAD: Lung adenocarcinoma; qRT-PCR: Quantities reverse transcription-polymerase chain reaction; OS: Overall survival; PFS: Progression-free survival; NA: Not available

**Table 2:** Association between high levels of lncRNAs and characteristics of patients with NSCLC

Characteristics	Studies	Case number	Pooled OR(95% CI)	P	Heterogeneity		References
					I2	P	
Gender (male vs female)	23	2111	1.00 (0.83,1.19)	0.96	0%	0.63	[5, 16, 17, 19, 20, 22–28, 30, 32–40, 46]
Smoking History (never vs ever)	16	1401	0.91 (0.73, 1.14)	0.43	42%	0.04	[5, 16, 17, 19, 20, 22, 23, 25, 30, 32–34, 36, 38–40]
Histological classification (LSCC vs LAD)	17	1634	1.03 (0.84, 1.26)	0.77	39%	0.05	[16, 17, 19, 20, 22–27, 30, 32–34, 36, 37, 46]
Tumor size (≤3 vs >3)	6	519	0.45 (0.17, 1.25)	0.13	85%	<0.001	[16],[25],[33],[36],[40],[41]
(≤5 vs >5)	6	529	0.61 (0.18, 2.01)	0.42	90%	<0.001	[17],[19],[23],[28],[35],[39]
TNM stage (I-II vs III-IV)	11	1091	0.63 (0.28, 1.44)	0.27	89%	<0.001	[16, 17, 19, 20, 22, 25, 32, 34, 36–38]
Lymph node metastasis (presence vs absence)	22	2036	1.70 (1.03, 2.80)	0.04	85%	<0.001	[5, 16, 17, 19, 20, 22, 24–28, 30, 32–40, 46]

LSCC: Lung squamous cell carcinoma; LAD: Lung adenocarcinoma; TNM: Tumor node metastasis; I^2^>50% with the random-effects model; I^2^<50% with the fixed-effects model.

### Prognosis

**Figure 2:**
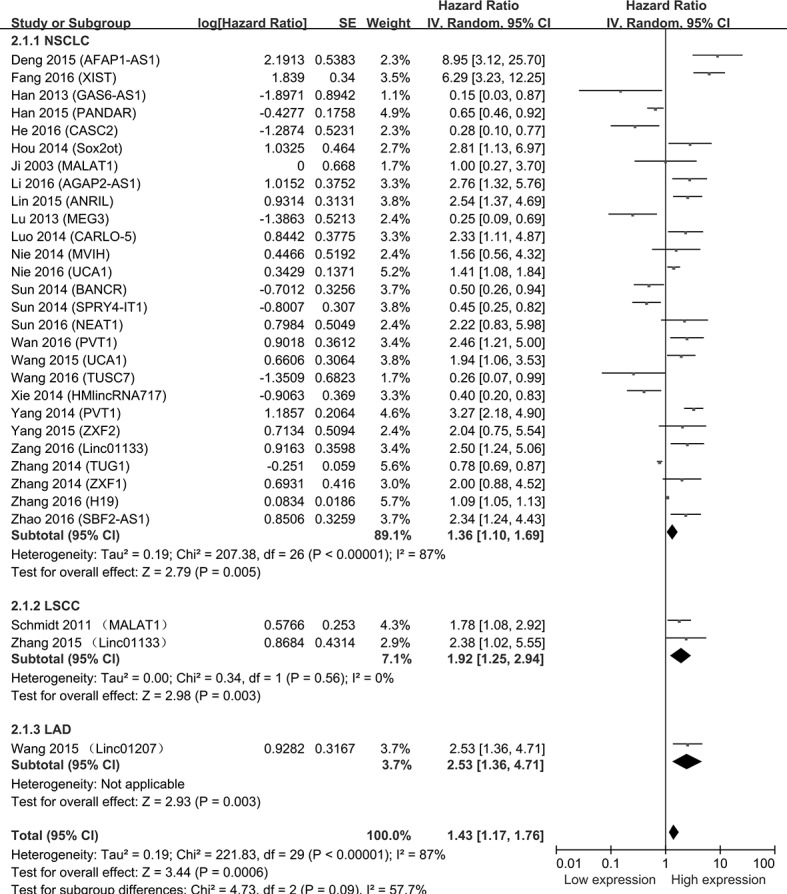
Forest plot of studies evaluating hazard ratios of lncRNAs expression and the overall survival in NSCLC The point estimate is bounded by a 95% confidence interval, and the perpendicular line represents no increased risk for the outcome. NSCLC: Non-small cell lung cancer; LSCC: Lung squamous cell carcinoma; LAD: Lung adenocarcinoma.

A total of 30 studies were reported that the expression levels of lncRNAs were related to OS. Hazard ratios (HRs) and 95% confidence interval (95% CI) were extracted from these studies. Analysis showed that the expression levels of lncRNAs were associated with the OS of NSCLC patients (HR = 1.43, 95%CI = 1.17-1.76, *P* < 0.001, random-effect) (Figure 2). Because 2 articles reported the relationship between lncRNAs and LSCC patients’ OS (HR = 1.92, 95%CI = 1.25-2.94, *P* = 0.003, fixed-effect) and 1 article was about LAD (HR = 2.53, 95%CI = 1.36-4.71, *P* = 0.003, fixed-effect) in the enrolled 30 studies, we performed the subgroup analysis. From the forest plot, the increased expressions of AFAP1-AS1, XIST, Sox2ot, AGAP2-AS1, ANRIL, CARLO-5, MVIH, UCA1, NEAT1, PVT1, ZXF2, Linc01133, ZXF1, H19, SBF2-AS1, MALAT1 and Linc01207 were associated with poor prognosis. Besides, GAS6-AS1, PANDAR, CASC2, MEG3, BANCR, SPRY4-IT1, TUSC7, HMlincRNA717 and TUG1 were correlated to poor prognosis with the decreased expression of lncRNAs in NSCLC.

**Figure 3:**
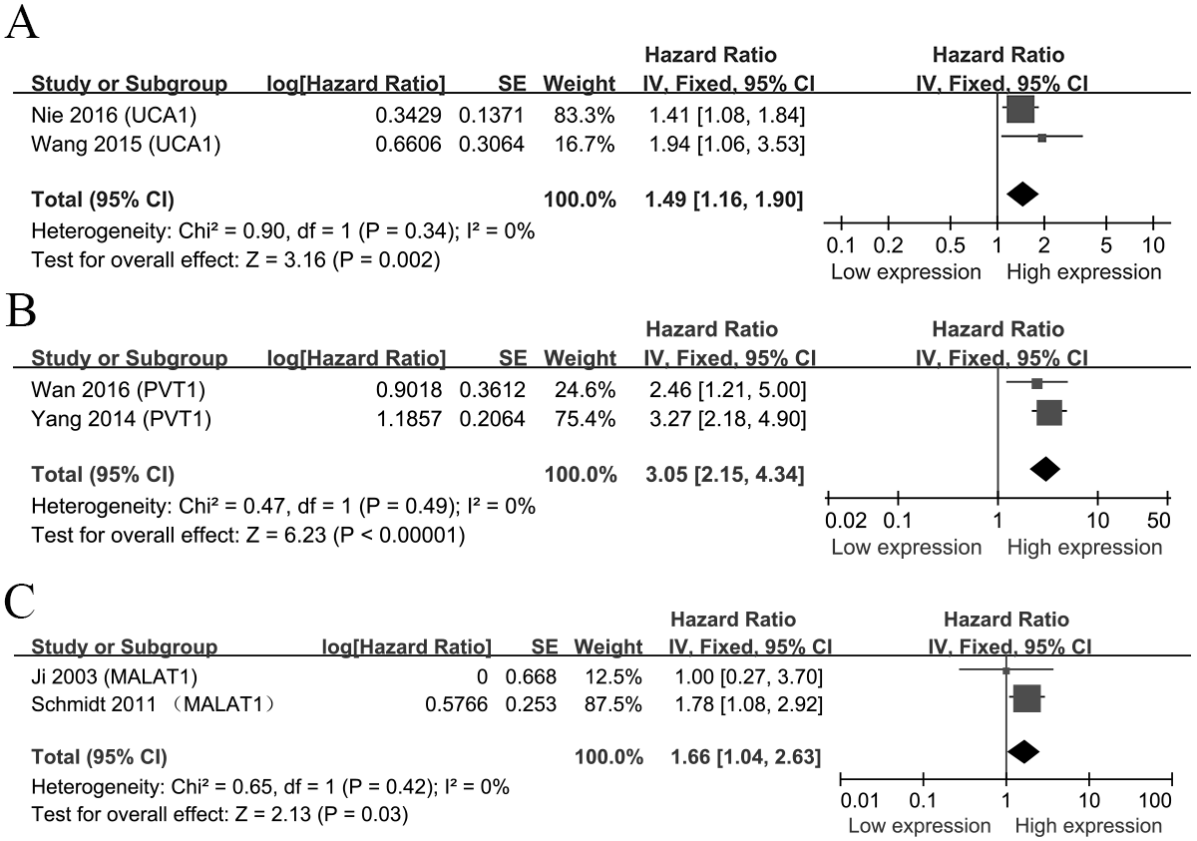
Forest plots of studies evaluating hazard ratios of up-regulated lncRNAs and the overall survival of NSCLC patients **A**. UCA1; **B**. PVT1. **C**. MALAT1.

In the enrolling studies, UCA1, PVT1 and MALAT1 were investigated in two researches, other lncRNAs were performed in single study. Then we conducted a meta-analysis on the relationship between the expressions of UCA1/PVT1/MALAT1 and the OS of patients with NSCLC. We found that the high levels of UCA1 were associated with a poor OS (HR = 1.49, 95% CI = 1.16-1.90, *P* = 0.002, fixed-effect) (Figure 3A). Meanwhile, a poor prognosis in NSCLC was found in the increased levels of PVT1 and MALAT1 (PVT1: HR = 3.05, 95% CI = 2.15-4.34, *P* < 0.001, fixed-effect) (MALAT1: HR = 1.66, 95% CI = 1.04-2.63, *P* = 0.03, fixed-effect) (Figure 3B-3C).

**Figure 4:**
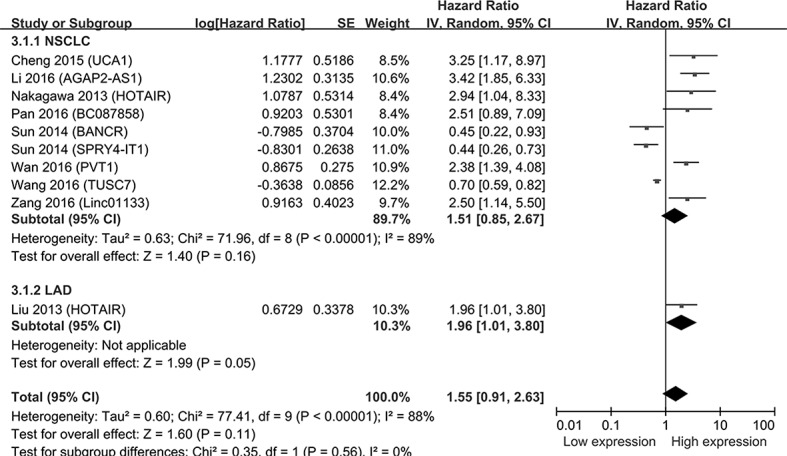
Forest plot of studies evaluating hazard ratios of lncRNAs expression and the progression-free survival in NSCLC The point estimate is bounded by a 95% confidence interval, and the perpendicular line represents no increased risk for the outcome. NSCLC: Non-small cell lung cancer; LAD: Lung adenocarcinoma.

10 studies enrolling 807 patients indicated that lncRNAs expression was related to the PFS in NSCLC. The up-regulated expression of UCA1, AGAP2-AS1, BC087858, HOTAIR, PVT1 and Linc01133 had a relatively poor prognosis, the decreased expressions of BANCR, SPRY4-IT1 and TUSC7 were related to poor prognosis. 1 study demonstrated that the relationship between the levels of lncRNA and LAD (HR = 1.96, 95%CI = 1.01-3.80, *P* = 0.05, fixed-effect). However, the expression of lncRNAs was not associated with the patients’ PFS in the enrolling studies (HR = 1.55, 95% CI = 0.91-2.63, *P* = 0.11, random-effect) (Figure 4).

### Correlation of lncRNAs with clinicopathological characteristics

As shown in Table 2, we performed a meta-analysis to evaluate the relationship between the transcription levels of lncRNAs and clinicopathological characteristics of patients with NSCLC. Odds ratios (OR)>1 stated that high levels of lncRNAs might be a risk factor in the features. Our results demonstrated that the high lncRNA expressions were significantly related to LNM (OR = 1.70, 95% CI = 1.03-2.80, *P* = 0.04, random-effect). Unfortunately, there was no correlation in gender (OR = 1.00, 95% CI = 0.83-1.19, *P* = 0.96, fixed-effect), smoking history (OR = 0.91, 95% CI = 0.73-1.14, *P* = 0.43, fixed-effect), histological classification (OR = 1.03, 95% CI = 0.84-1.26, *P* = 0.77, fixed-effect), tumor size (≤3 cm vs >3 cm: OR = 0.45, 95% CI = 0.17-1.25, *P* = 0.13, random-effect) (≤5 cm vs >5 cm: OR = 0.61, 95% CI = 0.18-2.01, *P* = 0.42, random-effect), TNM (OR = 0.63, 95% CI = 0.28-1.44, *P* = 0.27, random-effect) (Supplementary Figure 1).

### Publication bias and sensitivity analysis

**Figure 5:**
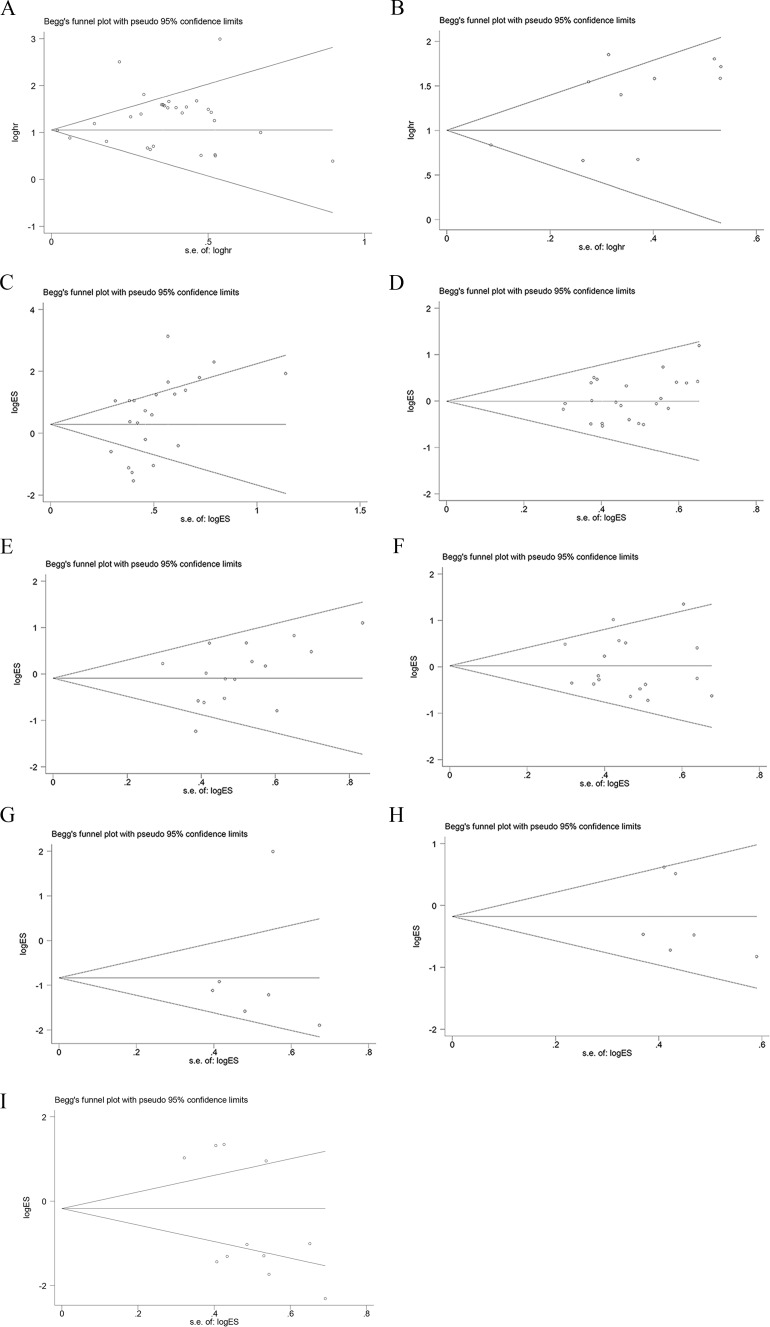
Begg’s test for publication bias **A**. overall survival; **B**. progression-free survival; **C**. lymph node metastasis; **D**. gender; **E**. smoking history; **F**. histological classification; **G**. tumor size (≤3 *vs* >3); **H**. tumor size (≤5 *vs* >5); I. TNM stage.

**Figure 6:**
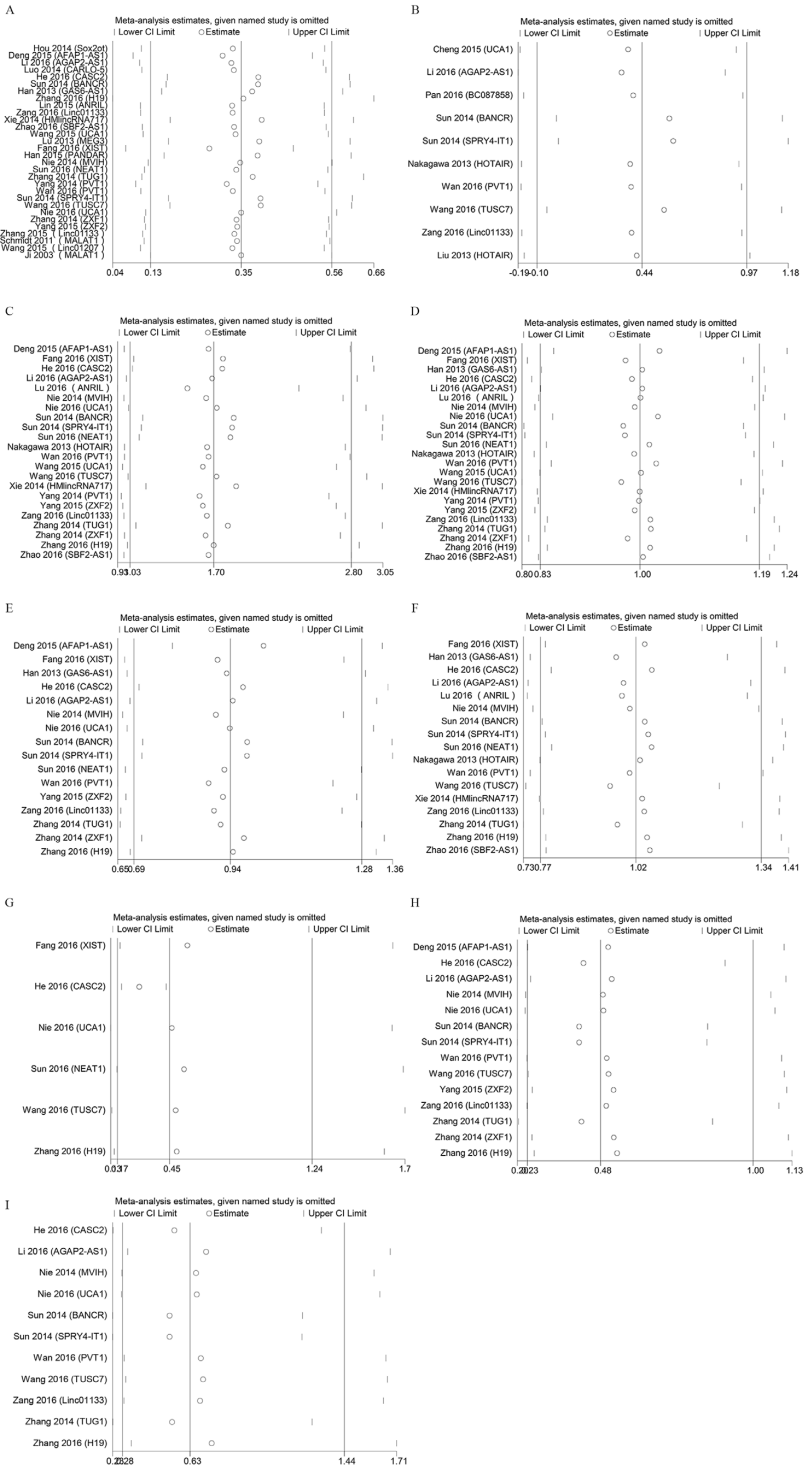
Sensitivity analyses of the studies **A**. overall survival; **B**. progression-free survival; **C**. lymph node metastasis; **D**. gender; **E**. smoking history; **F**. histological classification; **G**. tumor size (≤3 *vs* >3); **H**. tumor size (≤5 *vs* >5); **I**. TNM stage.

Begg’s test was used to evaluate the publication bias, respectively (Figure 5). In our meta-analysis, Begg’s test indicated there were no publication bias in all groups, due to all the values of *P*>0.05. We used Stata11.0 software to perform sensitivity analysis to assess whether the individual studies affected the overall results. The results indicated that individual study had little influence on our final results (Figure 6), and demonstrated that our analysis was relatively stable and credible.

## DISCUSSION

NSCLC is one of the leading causes of cancer-related death[33]. Identification of early disease indicators for prognosis is urgently required. Recently, mounting evidence demonstrated that lncRNAs dysregulation was involved in cancers [36]. H19 which was a direct target of oncogene c-Myc was an independent predictor for OS, as well as the TNM stage [16]. TUG1 was an important prognostic factor for NSCLC patients. In addition, knockdown of TUG1 was found to result in anti-apoptotic activity in NSCLC [34]. For cancer researches, most of cancer associated lncRNAs could be effective prognostic biomarkers and even therapeutic targets[43]. Therefore, we performed a meta-analysis to evaluate the association between lncRNAs and prognosis of patients with NSCLC.

Accumulating studies elaborated that the association between lncRNAs and NSCLC. In 2013, Lu *et al* [29] suggested that MEG3 could be considered as a negative prognostic factor for NSCLC patients and an indicative of poor survival rates. Meantime, the decreased levels of CASC2 predicted a poor prognosis and could be as an independent predictor for OS [22]. In our meta-analysis, we assessed the prognostic role of lncRNAs in NSCLC. Our results indicated that high expressions of lncRNAs represented a risk factor for OS in NSCLC. From these results, lncRNAs could be as biomarkers for the prognosis of NSCLC. Previous study indicated that TUSC7 acted as a growth suppressor in NSCLC through inhibiting the p21/p27, and low TUSC7 expression had a shorter PFS. Unfortunately, there was no relationship between lncRNAs expression and PFS in our meta-analysis. Therefore, further large-scale studies should be conducted.

In our meta-analysis, UCA1, PVT1 and MALAT1 were detected in two researches, and with the high levels of the three lncRNAs, the prognosis rate was low in NSCLC. UCA1 is originally identified in bladder transitional cell carcinoma. In 2016, Nie *et al* [38] revealed that UCA1 could promote NSCLC progression through functioning as miR-193a-3p sponge. Meanwhile, they found that UCA1-miR-193a-3p-ERBB4 signaling pathway might participate in the development of NSCLC. PVT1 is increased in the NSCLC patients, and the high expression of PVT1 was related with the poor prognostic outcome of cancer patients. Furthermore, PVT1 could decrease the levels of large tumor suppressor kinase 2 (LATS2), which could inhibit the growth and motility in NSCLC cells. Researchers also demonstrated that PVT1/EZH2/LATS2 could be considered as a new therapeutic target for patients with NSCLC [36]. Li *et al* [15] found that MALAT1 inhibited miR-204 expression and promoted the progress of metastasis. Our results indicated that these three lncRNAs had a significantly prognostic value in NSCLC.

Previous researches indicated that the expressions of lncRNAs were related to clinicopathological parameters in NSCLC, including TNM[30], tumor size[16], LNM[28] and some other features. However, in our meta-analysis, we only found that there was a relationship between the high level transcription of lncRNAs and LNM. In 2016, Sun *et al* [33] found that NEAT1 were associated with the TNM stage and tumor size in patients with NSCLC. The clinical pathological features of NSCLC patients revealed a significant association between increased MVIH expression and advanced pathological stage, LNM, and NSCLC tumor size. What is more, MVIH could promote cell proliferation and invasion in NSCLC cells [32]. Therefore, the possible reason for the uncorrelation between lncRNAs and other characters was the insufficient study for each lncRNA.

It should be stressed the limitations in our analysis. Most studies reported positive results, but those with negative results were generally less likely to be published. Then, the enrolling studies were only English researches, no other languages. In addition, there were insufficient data to fully confirm the association between lncRNAs and clinicopathological characteristics, which needs more studies. What is more, most of the population in our studies were Chinese, which also might affect the results. Recently, circulating lncRNAs were detected to evaluate their diagnostic significance in NSCLC[49]. In 2016, Liang *et al*[49] indicated that GAS5 was decreased in NSCLC plasma and could be considered as a biomarker for the diagnosis of NSCLC. However, due to the presence of RNases, circulating RNA has been thought to be unstable, especially in cancer patients [50]. Meantime, the diagnostic studies about lncRNAs are not enough. Therefore, another limitation was the lack of diagnosis about lncRNAs in our meta-analysis.

In summary, our meta-analysis was firstly to evaluate the expressions of lncRNAs and clinical values of patients with NSCLC. Furthermore, the result indicated that the level transcription was related to LNM. Despite these limitations, there was a relationship between lncRNAs levels and OS in NSCLC, which demonstrated that lncRNAs could be potential prognostic markers for NSCLC. However, large-scale and comprehensive researches were needed to illuminate our results.

## MATERIALS AND METHODS

### Publication search

We searched the databases PubMed, Embase and Web of Science for studies published up to October 2016 to obtain relevant articles for the meta-analysis. The search strategy used both medical subject heading terms and free-text words to increase the sensitivity of the search. We mainly searched three key aspects “lncRNA”, “cancer”, and “lung”, the detail search strategy was following: “Long noncoding RNA”, “lncRNA”, “LincRNA”, “Long intergenic non-coding RNA” and “NSCLC”, “non-small cell lung cancer“, “LAD”, “lung adenocarcinoma”, “LSCC”, “lung squamous cell cancer”. Meanwhile, the article covered was limited to human and English.

### Inclusion and exclusion criteria

In this meta-analysis, eligible studies had to meet the following standards: patients in the study were diagnosed with lung cancer; researches were association between lncRNAs and lung cancer; the prognostic value of lncRNAs was investigated; the relationship between lncRNAs expression and overall survival or progression-free survival was performed; sufficient published data were provided to calculate hazard ratios and 95% confidence interval. If there were duplicated data, we chose the most complete data or the most recent one. Exclusion criteria were as follow: studies without usable or insufficient data; case reports, reviews, letters and conference abstracts.

### Data extraction

Two investigators extracted relevant data from the eligible studies independently, including the first author, year of publication, country, the LncRNAs, tumor type, method, case number, cut-off value and follow-up months.

### Statistical analysis

HRs and 95% CIs were used to assess the association between lncRNAs and survival in NSCLC. An observed HR>1 implied a worse survival for the group with elevated lncRNAs expression. Conversely, HR < 1 implied a worse survival for the group with decreased lncRNAs expression [51]. Meanwhile, Odds ratios and 95% CI were used to evaluate the relationship between lncRNAs and clinicopathological features in these inclusive articles. The features included gender, lymph node status, tumor sizes, histological classification, smoking history and TNM. We used Revman5.3 Software (Revman, the Cochrane Collaboration) to perform the meta-analysis and evaluate heterogeneity between studies by Cochrane Q-test and P-values. If heterogeneity was present (*I^2^*≥50% or *P*≤0.05), random-effect model was used to calculate pooled HRs or ORs. If not, the fixed-effect model was more appropriate [52, 53]. The Stata11.0 Software (Stata, College Station) was performed to evaluate the sensitivity and publication bias of the studies. Publication bias was evaluated by Begg’s test, *P* < 0.05 was considered statistically significant.

## SUPPLEMENTARY FIGURE


