# Supplementary material for: The prognostic significance of long noncoding RNAs in non-small cell lung cancer: a meta-analysis
Source: Oncotarget. 2016 Dec 15;8(3):3957–68. doi: 10.18632/oncotarget.13956 (PMC5354806; doi:10.18632/oncotarget.13956)
Supplement: Supplementary file 1 [file oncotarget-08-3957-s001.pdf]

The prognostic significance of long noncoding RNAs in non-small cell lung cancer: a meta-analysis

Supplementary Material

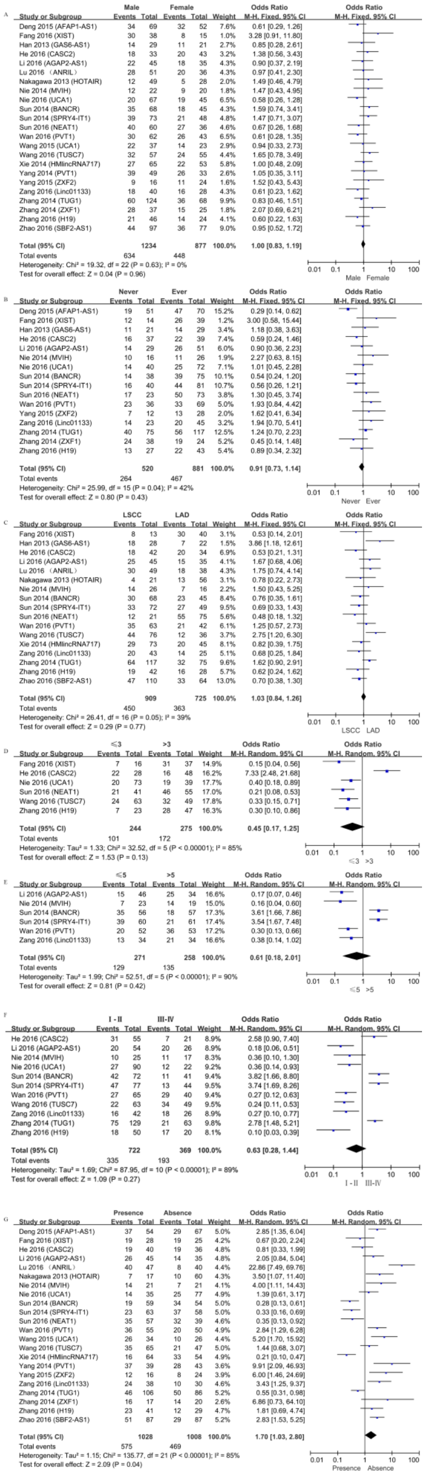

Supplementary Fig.1 Forest plot for the association between the high levels of lncRNAs with characteristics of patients with NSCLC. A. gender; **B.** smoking history; **C.** histological classification; **D.** tumor size (≤3 *vs* >3); **E.** tumor size (≤5 *vs* >5); **F**. TNM stage. **G.** lymph node metastasis.
